# Smoking Is Associated with More Abdominal Fat in Morbidly Obese Patients

**DOI:** 10.1371/journal.pone.0126146

**Published:** 2015-05-15

**Authors:** Raquel Chatkin, José Miguel Chatkin, Lucas Spanemberg, Daniela Casagrande, Mario Wagner, Cláudio Mottin

**Affiliations:** 1 Center of Obesity and Metabolic Syndrome, Hospital São Lucas da Pontifícia Universidade Católica do Rio Grande do Sul, Porto Alegre, Brazil; 2 Post-Graduation Program in Medicine and Health Science, School of Medicine Pontifícia Universidade Católica do Rio Grande do Sul, Porto Alegre, Brazil; 3 Department of Psychiatry, Hospital São Lucas da Pontifícia Universidade Católica do Rio Grande do Sul, Porto Alegre, Brazil; Medical University Innsbruck, AUSTRIA

## Abstract

**Introduction:**

While the association between cigarette smoking and abdominal fat has been well studied in normal and overweight patients, data regarding the influence of tobacco use in patients with morbid obesity remain scarce. The aim of this study is to evaluate body fat distribution in morbidly obese smokers.

**Methods:**

We employed a cross-sectional study and grouped severely obese patients (body mass index [BMI] >40 kg/m^2^ or >35 kg/m^2^ with comorbidities) according to their smoking habits (smokers or non-smokers). We next compared the anthropometrical measurements and body composition data (measured by electric bioimpedance) of both groups. We analyzed the effect of smoking on body composition variables using univariate and multiple linear regression (MLR); differences are presented as regression coefficients (b) and their respective 95% confidence intervals.

**Results:**

We included 536 morbidly obese individuals, 453 (84.5%) non-smokers and 83 (15.5%) smokers. Male smokers had a higher BMI (b=3.28 kg/m^2^, p=0.036), larger waist circumference (b=6.07 cm, p=0.041) and higher percentage of body fat (b=2.33%, p=0.050) than non-smokers. These differences remained significant even after controlling for confounding factors. For females, the only significant finding in MLR was a greater muscle mass among smokers (b=1.34kg, p=0.028). No associations were found between tobacco load measured in pack-years and anthropometric measures or body composition.

**Discussion:**

Positive associations between smoking and BMI, and waist circumference and percentage of body fat, were found among male morbidly obese patients, but not among females. To the best of our knowledge, this study is the first investigation of these aspects in morbidly obese subjects. We speculate that our findings may indicate that the coexistence of morbid obesity and smoking helps to explain the more serious medical conditions, particularly cardiovascular diseases and neoplasms, seen in these patients.

## Introduction

Non-communicable diseases (NCDs) are currently the leading global cause of death worldwide[1]. A large proportion of NCDs can be prevented if some of the principal risk factors are controlled, such as smoking and obesity. These risk factors are responsible for almost 80% of the deaths in this group of subjects and are mainly related to cardiovascular diseases, cancers, diabetes and chronic lung diseases[2].

Smoking and obesity are independent health risk factors but are also interrelated. However, this association remains not completely understood. The co-occurrence of these two clinical situations has devastating effects for human health[2]. According to Peeters et al[3], the life expectancy of obese smokers is 13 years less than normal weight non-smokers.

Smokers in general have lower body mass index (BMI) and lower body weight than non-smokers, paired by gender and age[4]. This is probably a consequence of several effects of nicotine, such as the increase in energy expenditure[2] and appetite suppression[5], among others. Smoking cessation is often associated with an increase in body weight, but such weight gain does not modify the benefits of stopping smoking[6,7,8].

Smoking is also associated with large waist circumference (WC), an indicator of abdominal obesity[2]. While high BMI is a strong predictor of mortality[9], abdominal obesity is a risk factor for obesity, morbidity and mortality[10,11].

Several demographic factors, such as level of education, age, and socioeconomic status, may interfere with the impact of smoking on body weight. In a previous study[12] we found that the greater the BMI, the greater is the risk of being a smoker, including among the morbidly obese subjects. However, Patel et al[13], studying low income subjects and patients with low educational level, found an inverse association between smoking and BMI, including a smaller prevalence of smokers in individuals with morbid obesity.

Indeed, it is less clear whether this known association of smoking and body weight also occurs in patients with morbid obesity. It is of critical importance to establish the role of smoking in changes of the body composition among morbidly obese patients. Various co-factors, such as age, gender, ethnic differences, tobacco load and socioeconomic status, have been shown to modify the effect of smoking on body weight and body shape[14,15] in these subjects, prompting further investigation. The higher risk of metabolic syndrome diseases[16] in morbid obese smokers is an example of a relevant association.

Therefore, while the association between cigarette smoking and body fat distribution has been well documented in normal and overweight patients, data addressing the influence of tobacco use on patients with morbid obesity remains scarce. The aim of this study was to evaluate the effect of current smoking comparing to non-smokers in body fat distribution, especially abdominal fat in these patients.

Considering what is already known for overweight and obese subjects, we hypothesized that morbidly obese subjects show different fat distributions according to their smoking status. This hypothesis was based on three sub-hypotheses: (1) morbidly obese smokers have a lower BMI than morbidly obese non-smokers; (2) morbidly obese smokers have more visceral fat than morbidly obese non-smokers; and (3) these results are maintained even after adjustment for confounding factors.

## Methods

### Participants and sampling

This is a cross-sectional study in which subjects were recruited from the *Centro da Obesidade e Síndrome Metabólica*, *Hospital São Lucas da Pontificia Universidade Católica do Rio Grande do Sul* (Obesity and Metabolic Syndrome Center: COM HSL-PUCRS) in Porto Alegre, Brazil.

Data were collected from January 2009 to December 2013. We included patients of both genders, 18 to 65 years of age, smokers or non-smokers and with BMI ≥40 kg/m^2^ or ≥35 kg/m^2^ with comorbidities[17]. The exclusion criteria were: any severe psychopathology, abusive use of alcohol or illegal drugs, pregnancy, breastfeeding and illiteracy.

All subjects gave written informed consent before entering the study and were grouped according to their smoking status. Current smokers were those subjects that have smoked ≥100 cigarettes in their lifetime and were still smoking daily or most of the days. Non-smokers were those who had never smoked or smoked <100 cigarettes in their lifetime and were not currently smoking. Smokers were also classified by the number of cigarettes smoked per day: mild (1–10 cigarettes/day), moderate (11–19 cigarettes/day) or severe (≥20 cigarettes/day) smokers[18,19].

Former smokers were defined as subjects who have quit smoking for at least 6 months before the interview date. They were included in the non-smokers group, in accordance with Caraballo et al.[20], the Canada Health Concerns: Terminology[18] and the Centers for Disease Control and Prevention[19]. Former smokers for less than 6 months were excluded from this study.

### Procedures

Patients were screened among those under evaluation for possible bariatric surgical procedure. Medical history and physical examination were always performed by a physician. A nutritionist (author RC) enrolled the patients into the study, and carried out anthropometric measurements (body weight and height, waist and hip circumference) and the procedures for body composition. For these latter procedures, the volunteer was required to be wearing light clothing and no shoes. Waist and hip circumferences were gauged with a tape (up to 3 meters). Waist circumference (WC) was measured to the nearest centimeter at the midpoint between the lower costal margin and the iliac crest. Hip circumference (HC) was measured at the greater prominence of the buttock. All these measurements were performed also by the first author (RC).

The body composition study included measurements of total body fat mass (kg), percentage of body fat (%), skeletal muscle mass/SMM (kg) and basal metabolic rate/BMR (kcal). Body composition assessments were performed by bioimpedance analysis (BIA) with segmented multifrequency tetrapolar and eight tactile electrodes (In Body 520—Biospace).

Blood samples were drawn for laboratory analysis using commercial kits. Lipid profile (total cholesterol, high density lipoprotein [HDL], low density lipoprotein [LDL], triglycerides), thyroid hormones (T3, T4 and thyroid-stimulating hormone [TSH]) and type 2 diabetes mellitus data (fasting blood glucose, glycemic index and glycated hemoglobin) were collected at the Hospital São Lucas Central Laboratory, according to standard parameters and methods.

A participant had diabetes when presenting fasting glucose levels of >126 mg/dL or >200 mg/dL 2 h after ingestion of glucose (75 g). The diagnosis of arterial hypertension was made for individuals with systolic blood pressure of ≥140 mmHg or diastolic blood pressure of ≥90 mm Hg. To be cataloged as dyslipidemic, the individual needed to have total cholesterol of ≥200 mg/dL, HDL <40 mg/dL (for males) or <50 mg/dL (for females), LDL >129 mg/dl or Triglycerides >150 mg/dL. Hypothyroidism was diagnosed when the TSH was >4.5 mIU/dL and T4 was <4.5 μg/dL[21].

The abuse of alcohol was considered when the subject stated consumption of greater than five cans of beer, one bottle of wine or three doses of liquor over a period of 3 h on 3 or more occasions[22]. The picker behavior was characterized when the volunteer had several small unplanned meals[23].

### Statistical analysis

We planned a study with non-smokers and smokers in a 4:1 inclusion ratio. Previous data[2],[12] indicated the probability of obesity among non-smokers as 0.2. If the true odds ratio for obesity in smoking relative to non-smoking subjects is 2.2, we would require 272 non-smokers and 68 smokers to have a statistical power of 0.8 and thus to be able to reject the null hypothesis at a significance level of (α) 0.05. To control for confounding and potential interaction effects we have increased our sample size by 50%.

Quantitative variables are described by means and standard deviations or by median and interquartile range when distributional assumptions were in doubt. Categorical variables are presented using absolute and relative frequencies. Student’s *t*-test was used to compare means and the Mann-Whitney test was employed to compare medians. We used the Pearson chi-squared test to compare proportions. In order to include corrections for multiple comparisons in the analysis of continuous variables, we used a multivariate ANOVA test with a Bonferroni correction.

To control for confounding factors, we used univariate and multiple linear regression analyzes. The effect of smoking on body composition variables was presented as regression coefficients (b) with their respective 95% confidence intervals.

The level of significance was set at 5% (p≤0.05) and analyses were performed using Statistical Package for the Social Sciences v. 22 (Chicago, IL, USA).

### Approvals

The study was approved by the Scientific Committee and by the Research Ethics Committee of the Pontificia Universidade Catolica do Rio Grande do Sul (PUCRS) under the number 234.527/2012.

## Results

A total of 536 individuals with morbid obesity were included and stratified according to their smoking status (i.e., as either smokers or non-smokers). The characteristics of the individuals included in the study are shown in Table 1.

**Table 1:** Clinical and demographic profile of the sample stratified by smoking status.

Variables	Total (n = 536)	Non-smokers (n = 453; 84.5%)	Smokers (n = 83; 15.5%)	p	p^multiple comparisons^
Gender female	384 (71.6%)	321 (70.9%)	63 (75.9)	0.427	-
Age, years	36.55 (±10.20)	36.45 (±10.31)	37.14 (±9.67)	0.568	0.724
Weight, Kg	122.48 (±22.52)	122.2 (±22.44)	126.5 (±22.70)	0.111	0.108
BMI, Kg/m^2^	43.65 (±6.18)	43.39 ± (6.18)	45.08 (±6.07)	**0.021**	**0.024**
WC, cm	128.36 (±13.66)	128.03 (±13.65)	130.18 (±13.67)	0.187	0.212
HC, cm	133.55 (±11.85)	133.2 (±12.05)	135.4 (±10.56)	0.109	0.135
WHR	0.964 (±0.09)	0.964 (±0.09)	0.964 ± (0.10)	0.987	0.997
SMM, Kg	33.90 (±7.17)	34.2 (±7.13)	34.8 (±7.42)	0.456	0.414
Body fat, Kg	62.55 (±11.94)	61.2 (±13.13)	64.9 (±12.93)	**0.020**	**0.021**
Body fat, %	49.78 (±4.35)	49.58 (±4.47)	50.85 (±3.45)	**0.004**	**0.016**
MBR, Kcal/dia	1690.02 (±257.9)	1688 (±259.65)	1699 (±249.39)	0.369	0.712
Diabetes	103 (19.2)	83 (18.3)	20 (24.1)	0.227	-
HOMA	415 (77.4)	350 (77.3)	65 (78.3)	0.887	-
Dyslipidemia	298 (55.6)	246 (54.3)	52 (62.7)	0.186	-
Hypertension	243 (45.3)	209 (46.1)	34 (41)	0.403	-
Hypothyroidism	95 (17.7)	82 (18.1)	13 (15.7)	0.643	-
Alcohol abuse	102 (19.1)	78 (17.3)	24 (28.9)	**0.022**	**-**
Picky eater	219 (40.9)	180 (39.7)	39 (47)	0.226	-
NpacY	-	-	11 (3 to 20)	-	-
N cigarettes /day	-	-	20 (8 to 20)	-	-
Age of onset	-	-	18 (16 to 22)	-	-
Smoking period, years	-	-	16 (8 to 22)	-	-

Note: Results expressed as mean ±SD: sex, age, weight, BMI (body mass index), WC (waist circumference), HC (hip circumference), WHR (waist to hip ratio), SMM (skeletal muscle mass); body fat, body fat, MBR (basal metabolic rate); Results expressed in number (percentage): diabetes, HOMA (homeostasis model assessment insulin resistant), dyslipidemia, hypertension, hypothyroidism, alcohol abuse, picky eater; Results expressed as median (25/75 percentile): NpacY (number of pack-years), N cigarettes/day (number of cigarettes/day) age of onset, smoking period.

Discrete variables analyzed by Pearson or Fisher chi-square test; continuous variables analyzed by Student t test and ANOVA test with Bonferroni correction (for multiple comparisons).

Values in bold = statistic significant (p<0.05).

The majority of the subjects was non-smokers (n = 453; 84.5%), which had a significantly lower mean BMI than the smokers (43.4 kg/m^2^ *vs*. 45.1 kg/m^2^ respectively; p = 0.021). There were significant differences between these two groups for mean fat weight (61.2 kg *vs*. 64.9 kg for non-smokers and smokers, respectively; p = 0.020) and mean fat percentage (49.6% *vs*. 50.9% for non-smokers and smokers, respectively; p = 0.004). Abusive use of alcohol was also significantly higher among smokers compared to non-smokers. No other significant differences were found among other clinical and sociodemographic variables.

Table 2 shows the participants’ clinical and demographic profiles according to smoking status and stratified by gender. After stratifying by gender, male smokers were heavier (151.9 kg *vs*. 140.2 kg; p = 0.036), had greater BMI (48.3 kg/m^2^ *vs*. 44.9 kg/m^2^; p = 0.029), larger WC (144.7 cm *vs*. 138.8 cm; p = 0.042), greater WHR (1.08 *vs*. 1.05; p = 0.015), lower body fat (65.9 kg *vs*. 74.7 kg; p = 0.022), and higher fat percentage (48.3% *vs*. 45.9%; p = 0.047). Among women, smokers had higher WHR (1.07 *vs*. 1.04; p = 0.020) but lower alcohol abuse (2.2% *vs*. 10.6%; p = 0.020). No other characteristics were found to have significant differences between female smokers and non-smokers.

**Table 2:** Clinical and demographic profile of the sample stratified by gender and according to smoking status.

Variables	Male				Female			
	Non Smoker (n = 132)	Smoker (n = 20)	p	p^multiple comparisons^	Non Smoker (n = 321)	Smoker (n = 63)	p	p^multiple comparisons^
Age, Years	36.37 (±10.02)	33.15 (±9.26)	0.178	0.178	36.48 (±10.43)	38.41 (±9.51)	0.174	0.262
Weight, Kg	140.22 (±23.00)	151.85 (±22.26)	**0.036**	**0.037**	114.24 (±17.25)	117.96 (±15.84)	0.113	0.115
BMI, Kg/m^2^	44.85 (±6.50)	48.30 (±6.68)	**0.029**	**0.024**	42.79 (±5.94)	44.06 (±5.55)	0.119	0.140
WC, cm	138.78 (±12.16)	144.70 (±11.21)	**0.042**	**0.042**	123.60 (±11.64)	125.57 (±10.91)	0.216	0.263
HC, cm	132.83 (±13.35)	136.40 (±14.18)	0.270	0.270	133.36 (±11.49)	135.17 (±9.25)	0.239	0.294
WHR	1.04 (±0.06)	1.06 (±0.09)	0.394	0.277	0.929 (±0.08)	0.931 (±0.08)	0.892	0.897
SMM, kg	42.11 (±5.73)	43.40 (±5.67)	0.351	0.298	30.37 (±4.24)	31.55 (±5.33)	0.051	**0.045**
Body fat, Kg	74.73 (±15.22)	65.87 (±16.10)	**0.022**	**0.022**	61.82 (±10.45)	62.31 (±53.78)	0.943	0.122
Body fat, %	45.91 (±5.05)	48.30 (±4.18)	**0.047**	**0.042**	51.10 (±3.15)	51.67 (±2.74)	0.184	0.203
MBR, kcal/day	1988.48 (±211.78)	2043.75 (±205.34)	0.277	0.277	1563.63 (±154.42)	1588.71 (±134.62)	0.234	0.234
Diabetes	34 (25.8)	9 (45.0)	0.108	-	49 (15.30)	11 (17.50)	0.704	-
HOMA	112 (84.8)	16 (80.0)	0.525	-	238 (74.10)	49 (77.8)	0.635	-
Dyslipidemia	79 (59.8)	12 (60.0)	1.00	-	167 (52.0)	40 (63.5)	0.099	-
Hypertension	70 (53.0)	11 (55.0)	1.00	-	139 (43.30)	23 (36.5)	0.333	-
Hypothyroidism	13 (9.8)	2 (10.0)	1.00	-	69 (21.50)	11 (17.5)	0.611	-
Alcohol abuse	44 (33,3)	10 (50.0)	0.209	-	34 (10.60)	14 (2.22)	**0.020**	**-**
Picky eater	46 (34,8)	7 (35.0)	1.00	-	134 (41.9)	32 (50.8)	0.212	-
NpacY	-	13.44 (±12.79)	-	-	-	16.55 (±17.61)	-	-
Number cigarettes/day	-	17.70 (±9.27)	-	-	-	15.41 (±12.27)	-	-
Age of starting, years	-	18.65 (±4.10)	-	-	-	19.89 (±5.83)	-	-
Smoking period, years	-	14.05 (±8.59)	-	-	-	24.55 (±59.19)	-	-

Note: Results expressed as mean (± standard deviation): sex, age, weight, BMI (body mass index), WC (waist circumference), HC (hip circumference), WHR (waist to hip ratio), SMM (skeletal muscle mass); body fat, percentage of body fat, MBR (basal metabolic rate), NpacY (number of pack-years), number of cigarettes/day, age of starting, smoking period; Results expressed in number (percentage): Diabetes, HOMA (homeostasis model assessment insulin resistant), dyslipidemia, hypertension, hypothyroidism, alcohol abuse, picky eater.

Discrete variables analyzed by Pearson or Fisher chi-square test; continuous variables analyzed by Student t test and ANOVA test with Bonferroni correction (for multiple comparisons).

Values in bold = statistic significant (p<0.05).

Table 3 presents the univariate and multiple analyses using linear regression to evaluate the effect of smoking in relation to anthropometric and bioimpedance data, stratified by gender.

**Table 3:** Univariate analysis and multiple linear regression to evaluate the effect of smoking in the anthropometric and bioimpedance outcomes, total sample and stratified by gender.

Outcomes	Total (n = 536)			Male (n = 152)			Female (n = 384)		
	b	CI 95%	p	b	95%CI	p	b	95%CI	P
BMI (kg/m2)									
Non-Adjusted	1.69	0.24 to 3.13	**0.022**	3.44	0.35 to 6.54	**0.029**	1.27	-0.33 to 2.86	0.119
Adjusted	1.96	0.54 to 3.39	**0.007**	3.28	0.21 to 6.34	**0.036**	1.46	-0.15 to 3.07	0.075
WC (cm)									
Non-Adjusted	2.15	-1.05 to 5.36	0.187	5.92	0.21 to 11.6	**0.042**	1.97	-1.16 to 5.09	0.216
Adjusted	3.22	0.46 to 5.97	**0.022**	6.07	0.24 to 11.9	**0.041**	2.24	-0.91 to 5.38	0.162
HC (cm)									
Non-Adjusted	2.27	-0.51 to 5.04	0.109	3.57	-2.81 to 9.96	0.270	1.82	-1.21 to 4.84	0.239
Adjusted	2.52	-0.20 to 5.24	0.070	3.16	-3.09 to 9.40	0.999	2.17	-0.84 to 5.17	0.157
WHR									
Non-Adjusted	0.00	-0.02 to 0.02	0.987	0.02	-0.01 to 0.05	0.277	0.00	-0.02 to 0.02	0.892
Adjusted	-0.01	-0.02 to 0.01	0.622	0.02	-0.01 to 0.05	0.167	0.00	-0.02 to 0.02	0.913
Basal Metabolic rate Kcal/dia									
Non-Adjusted	11.4	-49.4 to 72.3	0.712	55.3	-44.8 to 155	0.277	25.1	-16.2 to 66.4	0.234
Adjusted	32.6	-7.2 to 72.5	0.108	40.2	-61.9 to 142	0.438	28.8	-12.2 to 69.8	0.168
Fat percentage %									
Non-Adjusted	1.27	0.25 to 2.29	**0.015**	2.38	0.04 to 4.73	**0.047**	0.57	-0.27 to 1.41	0.184
Adjusted	1.11	0.23 to 1.99	**0.014**	2.33	0.00 to 4.67	**0.050**	0.58	-0.28 to 1.44	0.183
SMM (kg)									
Non-Adjusted	0.59	-1.09 to 2.28	0.490	1.29	-1.43 to 4.00	0.351	1.18	-0.02 to 2.39	0.054
djusted	1.25	0.13 to 2.38	**0.029**	0.85	-1.92 to 3.61	0.546	1.34	0.15 to 2.54	**0.028**

Note: Adjusted for gender (except on gender stratum), age, diabetes, dyslipidemia, alcohol abuse and picky eater; b = regression coefficient; BMI = body mass index; WC = waist circumference; HC = hip circumference; WHR = waist to hip ratio; SMM = Muscle mass; BMR = basal metabolic rate; values in bold = statistically significant (p<0.05)

Considering the whole sample, in the univariate analysis the BMI was found to be higher in morbidly obese smokers than in non-smokers (b = 1.69 kg/m^2^, 95%CI 0.24 to 3.13, p = 0.022), and this significant difference was maintained after adjusting for confounding factors (b = 1.96 kg/m^2^, 95%CI 0.54 to 3.39, p = 0.007). In the univariate analysis, smoking did not affect WC, but it became significant after adjustment (included factors were gender, age, diabetes mellitus, dyslipidemia, alcohol abuse and picker behavior). Thus, obese smokers had on average 3.22 cm (95%CI 0.46 to 5.97, p = 0.022) greater WC than obese non-smokers. Also, smokers had higher percentage of fat (b = 1.11%, 95%CI 0.23 to 1.99, p<0.014) and weight of lean mass (SMM) (b = 1.25Kg, 95%CI 0.13 to 2.38, p = 0.029) compared to non-smokers after adjustment for the same confounding factors.

When these analyses were stratified by gender and controlled by the others confounding factors, male smokers presented higher BMI (b = 3.28 kg/m^2^, 95%CI 0.21 to 6.34, p = 0.036), showed 6.07 cm larger WC (95%CI 0.24 to 11.9, p = 0.041), and presented higher fat percentage (b = 2.33%; 95%CI 0.00 to 4.67, p = 0.050). Among women, the only significant difference observed after adjustment was a higher SMM (b = 1.34Kg 95%CI 0.15 to 2.54 p = 0.028) for smokers. The variables HC, WHR, and BMR showed no significant difference for both genders.

We also performed the same univariate and multiple linear regression analyses after removing the former smokers (n = 101) from the study group. The results for the total sample were similar, with smokers characterized by higher BMIs, larger WCs, and higher percentages of fat and lean mass weight than never-smokers. However, in this model, the stratification by gender did not reveal differences between smokers and non-smokers in males and only maintained the greatest SMM among female smokers (S1 Table).

Regarding the number of cigarettes smoked per day among the 83 smoking subjects, 39 (47.0%) subjects were considered mild smokers, 35 (42.2%) moderate smokers, and 9 (10.8%) severe smokers. The median of the smoking period was 16 (8 to 22) years, the age of starting smoking was 18 (16 to 22) years-old and the median pack-years for the whole sample was 11 (3 to 20). There were no significant differences between genders among these variables (S2 Table). We also found a non-significant effect of the smoking load (measured by pack-years) upon anthropometric and bioimpedance variables, even after controlling for confounding factors (S3 Table).

## Discussion

In this study we found that smoking has a significant effect on anthropometric and bioimpedance outcomes in morbidly obese subjects, and that this effect differs according to gender. To our knowledge, this is the first study to apply traditional anthropometric tools together with a more accurate instrument (e.g., BIA) to evaluate the effects of smoking on body composition within this special patient group.

Considering the whole sample, smoking was significantly associated with higher BMI, larger WC, greater fat percentage and SMM, even after adjusting to several confounding factors (gender, age, diabetes mellitus, dyslipidemia, alcohol abuse and picker behavior). Some of these findings have already been described for obese and non-obese individuals[24,25,26,27,28,29], but not for morbidly obese subjects.

When these outcomes were stratified according to gender and after adjustments for several confounding factors, male smokers showed significantly higher BMI, larger WC, and greater fat percentage compared to male non-smokers. Meanwhile, the only significant difference observed among female smokers was a greater SMM.

There are several possible explanations for these results. Our findings might be related to the differences in how sex hormones interact with fat distribution. As a consequence, central adiposity is more likely in men while gluteal fat is more usual in woman[28,30]. Sedentary behavior is another possible explanation for the observed gender differences in WC and adiposity. Although morbidly obese individuals have more sedentary behaviors than non-obese individuals, men are more likely to do less exercises and eat more snacks, drink more beverages and spend more time watching TV than women. This behavior could contribute to the significantly higher accumulation of abdominal fat in males compared to females[31].

Lemieux et al[32] have previously shown that men and women have the same increase in total body fat mass and store equal amounts of adipose tissue in the abdominal subcutaneous compartment. However, they suggest that men accumulate more adipose tissue in visceral depots than women, while women store more fat tissue in specifics regions (e.g., gluteofemoral).

In addition, the higher serum levels of adiponectin in females compared to males[33] might also account for this finding. The adiponectin hormone is produced by fat tissue and has important roles in several clinical situations, such as systemic arterial hypertension, atherosclerotic events and type 2 diabetes. Adiponectin also acts on metabolic rate, oxidative stress and inflammatory response[34,35]. Therefore, the higher serum levels of adiponectin in women could be protective for abdominal fat accumulation. Nevertheless, there is lower production of adipokines in obese individuals compared to non-obese individuals, but such production increases when these obese individuals lose weight[33].

Unlike the reports regarding normal and obese individuals, which have shown a lower BMI in smokers compared to non-smokers[27,36,37,38], we found the opposite in smoking men with morbid obesity. Among our subjects, smoking was associated with increased abdominal obesity in morbidly obese male smokers and the higher BMI described here could be a direct consequence. Similar results are found in the literature[24,25,26,27]. Thus, the typical effects of smoking—decreased appetite[5] and increased energy expenditure[2] leading to decreased BMI and body weight-, in this group of patients might be compensated and even overcome by the effect of smoking on body composition, increasing central obesity and subsequently increasing BMI. Among females, we found only a significant difference in skeletal muscle mass between the smokers and non-smokers.

In order to compare our findings with a larger dataset, we analyzed data from The National Health and Nutritional Examination Survey (NHANES, 2011–2012)database, including just patients with BMI ≥35 kg/m^2^ according to smoking status defined by cotinine measurement (smokers defined by cotinine >15ng/mL and former smokers included in the non-smoking group). Morbidly obese smokers and non-smokers presented similar BMI values among males (n = 297; 41.05 kg/m^2^ vs. 39.95 kg/m^2^, p = 0.212) and females (n = 484; 41.37 kg/m^2^ vs. 40.67 kg/m^2^, p = 0.693). When we analyzed just those individuals with BMI ≥40 kg/m^2^, smokers showed a higher but non-significant BMI than non-smokers for males (n = 111; 46.97 kg/m^2^ vs. 44.66 kg/m^2^, p = 0.148) and females (n = 225; 46.13 kg/m^2^ vs. 44.89 kg/m^2^, p = 0.294). We found a significantly higher BMI in smokers compared with non-smokers in males (48.30 kg/m^2^ vs. 44.85 kg/m^2^, p = 0.029), but not in females (44.06 kg/m^2^ vs. 42.79 kg/m^2^, p = 0.119). Despite the differences between the samples (our sample derives from a clinical group, with a predominance of class III obesity, and the NHANES sample includes communitarian subjects, with a predominance of class II obesity), these results show a similar tendency of a slightly higher BMI mainly in male morbidly obese smokers.

High percentage of visceral fat is a risk factor associated with unhealthy lifestyle and active smoking seems to be a part of such risky behavior[39,40]. Greater amounts of visceral fat are associated with metabolic syndrome[28,29], type-2 diabetes[41,42], hyperlipidemia and hypertension[43]. Some studies have also shown a direct association between smoking and increased risk for metabolic syndrome[39,44], and that this is related to the number of cigarettes smoked per day [45].

Thus, while morbid obesity *per se* is associated with a substantial increase in morbidity and mortality[46,47], smoking may add a significant burden, especially for morbidly obese patients. Freedman et al.,[48] in a large prospective study, found that obesity combined with current smoking was associated with higher risks for circulatory diseases and mortality in people aged less than 65 years compared to never smokers with normal weight. What seems probable is that the mortality risk among obese smokers, even young obese smokers, far exceeds the sum of the individual risks related to morbid obesity and smoking[49].

The number of smoked cigarettes per day is also associated with larger WC[36,50], WHR[51,52] and BMI[51,52,53], with severe smokers showing higher measures of these parameters. In our sample, however, we found no significant association between pack-years or number of smoked cigarettes per day with body fat. A possible explanation for this finding might be the low number of smokers in our sample, particularly severe smokers. Thus, such a small group could have a conservative effect in these parameters, reducing the chance of a positive association.

This study has some limitations. First, it is a cross-sectional study that does not allow us to infer about cause and effect. Second, there was no chemical verification of smoking status. Although this may be a problem, there are studies showing that self-reported smoking status are reliable in special groups of subjects.[20] Considering the possibility of a near surgery and the fear of complications, we believe that this information in this group of patients can be trusted. Another point is that we had a greater number of female smokers in our sample. While most studies have shown that the prevalence smokers is higher in males than females[54,55,56], among morbidly obese subjects this may not be true. A similar finding to ours was described by Koster et al.,[54] who also shown that the percentage of smoking women with BMI >35 kg/m^2^ was greater than among males[25,54,55,56]. Extracted and analyzed data from NHANES database registered that obese subjects with BMI >35 kg/m^2^ had a higher (but non-significant) percentage of male smokers (21.2 *vs*. 17.4%, p = 0.181). In our study, we found smoker frequencies of 13.20% and 16.40% among males and females, respectively (p = 0.349). The gender differences in these both studies were not significant. Our findings can be also explained by the fact that our sample consisted of candidates for bariatric surgery, where the demand for the procedure is higher among women[55,56,57,58] and by the higher number of obese women in the world[59,60,61].

Several strengths of this study should also be highlighted. We used an accurate method to measure the percentage of body fat, validated in 2014 by Faria et al[62]. All anthropometric measures were collected by nutritional experts. Furthermore, this was the first study evaluating the association of body shape and smoking in a large sample of morbidly obese patients.

## Conclusions

Smoking significantly increases the anthropometric and body composition outcomes (WC and BMI, and percentage of body fat, respectively) among male morbidly obese subjects. Among females, the effect was significant only in muscle mass.

We partially confirmed our initial hypotheses, founding that morbidly obese male smokers have greater central adiposity compared to non-smokers. However, contrary to our original hypothesis, there is a gender difference in several of the studied outcomes. Morbidly obese male smokers had higher BMI, even after adjusting for confounding factors, but not in females.

The role of smoking on changes in body composition among morbidly obese patients seems to be significant and is probably crucial to understanding the mechanisms of disease in these subjects.

## Supporting Information

S1 Table Univariate analysis and multiple linear regression to evaluate the effect of smoking in the anthropometric and bioimpedance outcomes, sample stratified by gender and just with smokers and never-smokers (without former smokers). Note: Adjusted for gender (except on gender stratum), age, diabetes, dyslipidemia, alcohol abuse and picky eating; b = regression coefficient; BMI = body mass index; WC = waist circumference; HC = hip circumference; WHR = waist to hip ratio; SMM = muscle mass; BMR = basal metabolic rate; values in bold = statistically significant (p<0.05).
(DOCX)

S2 Table Comparison of smoking characteristics by gender. Note: MD = median.
(DOCX)

S3 Table Univariate analysis and multiple linear regression to evaluate the effect of pack-year units in the anthropometric and bioimpedance outcomes. Note: Adjusted for gender (except on gender stratum), age, diabetes, dyslipidemia, alcohol abuse and picky eater; b = regression coefficient; BMI = body mass index; WC = waist circumference; HC = hip circumference; WHR = waist to hip ratio; SMM = muscle mass; BMR = basal metabolic rate; values in bold = statistically significant (p<0.05).
(DOCX)
