# Supplementary material for: Smoking Is Associated with More Abdominal Fat in Morbidly Obese Patients
Source: PLoS One. 2015 May 15;10(5):e0126146. doi: 10.1371/journal.pone.0126146 (PMC4433108; doi:10.1371/journal.pone.0126146)
Supplement: S1 Table — Note: Adjusted for gender (except on gender stratum), age, diabetes, dyslipidemia, alcohol abuse and picky eating; b = regression coefficient; BMI = body mass index; WC = waist circumference; HC = hip circumference; WHR = waist to hip ratio; SMM = muscle mass; BMR = basal metabolic rate; values in bold = statistically significant (p<0.05). (DOCX) [file pone.0126146.s001.docx]

**S1 Table:**

| Outcomes | Total (n=435) | | |  | Male (n=124) | | |  | Female (n=311) | | |
| --- | --- | --- | --- | --- | --- | --- | --- | --- | --- | --- | --- |
|  | b | CI 95% | p |  | b | 95%CI | p |  | b | 95%CI | P |
| BMI (kg/m2) |  |  |  |  |  |  |  |  |  |  |  |
| Non-Adjusted | 1.62 | 0.19 to 3.04 | 0.026 |  | 2.94 | -0.14 to 6.03 | 0.061 |  | 1.40 | -0.12 to 2.93 | 0.072 |
| Adjusted | 1.90 | 0.50 to 3.30 | **0.008** |  | 2.75 | -0.34 to 5.84 | 0.081 |  | 1.47 | -0.09 to 3.04 | 0.065 |
| WC (cm) |  |  |  |  |  |  |  |  |  |  |  |
| Non-Adjusted | 2.44 | -0.73 to 5.61 | 0.132 |  | 5.14 | 0.35 to 10.63 | 0.066 |  | 2.76 | -1.14 to 5.68 | 0.063 |
| Adjusted | 3.53 | 0.92 to 6.14 | **0.008** |  | 4.96 | -0.63 to 10.55 | 0.082 |  | 2.86 | -0.10 to 5.83 | 0.059 |
| HC (cm) |  |  |  |  |  |  |  |  |  |  |  |
| Non-Adjusted | 1.88 | -0.88 to 4.65 | 0.181 |  | 2.40 | -4.08 to 8.88 | 0.465 |  | 1.76 | -1.21 to 4.74 | 0.245 |
| Adjusted | 2.53 | -0.22 to 5.29 | 0.071 |  | 2.35 | -4.08 to 8.80 | 0.470 |  | 1.97 | -1.02 to 4.96 | 0.196 |
| WHR |  |  |  |  |  |  |  |  |  |  |  |
| Non-Adjusted | 0.01 | -0.01 to 0.02 | 0.672 |  | 0.02 | -0.01 to 0.05 | 0.211 |  | 0.01 | -0.01 to 0.03 | 0.466 |
| Adjusted | 0.01 | -0.01 to 0.02 | 0.390 |  | 0.02 | -0.01 to 0.05 | 0.225 |  | 0.07 | -0.01 to 0.03 | 0.519 |
| Basal Metabolic rate Kcal/dia |  |  |  |  |  |  |  |  |  |  |  |
| Non-Adjusted | 12.1 | -49.8 to 74.2 | 0.700 |  | 45.2 | -54.9 to 145 | 0.373 |  | 31.6 | -7.35 to 70.60 | 0.111 |
| Adjusted | 37.9 | -1.09 to 76.9 | 0.057 |  | 33.2 | -69.0 to 135 | 0.521 |  | 35.4 | -3.28 to 74.09 | 0.073 |
| Fat percentage % |  |  |  |  |  |  |  |  |  |  |  |
| Non-Adjusted | 1.11 | 1.12 to 2.10 | **0.028** |  | 1.96 | -0.30 to 4.22 | 0.089 |  | 0.51 | -0.32 to 1.35 | 0.229 |
| Adjusted | 0.97 | 0.09 to 1.84 | **0.030** |  | 1.83 | -0.45 to 4.12 | 0.116 |  | 0.56 | -0.29 to 1.42 | 0.200 |
| SMM (kg) |  |  |  |  |  |  |  |  |  |  |  |
| Non-Adjusted | 0.65 | -1.07 to 2.37 | 0.458 |  | 1.12 | -1.48 to 3.83 | 0.413 |  | 1.37 | 0.21 to 2.54 | **0.020** |
| Adjusted | 1.50 | 0.38 to 2.61 | **0.008** |  | 0.78 | -1.97 to 3.53 | 0.576 |  | 1.57 | 0.40 to 2.73 | **0.008** |
