# Supplementary material for: Smoking Is Associated with More Abdominal Fat in Morbidly Obese Patients
Source: PLoS One. 2015 May 15;10(5):e0126146. doi: 10.1371/journal.pone.0126146 (PMC4433108; doi:10.1371/journal.pone.0126146)
Supplement: S2 Table — Note: MD = median. (DOCX) [file pone.0126146.s002.docx]

**S2 Table:**

| Variables | Total (n=83) | Male (n=20) | Female (n=63) | p |
| --- | --- | --- | --- | --- |
| Nº cigarettes/day; Md (P25 to P75) | 20 (8 to 20) | 20 (10 to 20) | 15 (6 to 20) | 0.190 |
| Nº cigarettes/group; n (%) |  |  |  | 0.714 |
| 1 to 10 | 39 (47.0) | 8 (40.0) | 31 (49.2) |  |
| 11 to 20 | 35 (42.2) | 10 (50.0) | 25 (39.7) |  |
| > 20 | 9 (10.8) | 2 (10.0) | 7 (11.1) |  |
| Smoking time (ys); Md (P25 to P75) | 16 (8 to 22) | 16 (8 to 25) | 15 (7 to 20) | 0.206 |
| Age of onset (ys); Md (P25 to P75) | 18 (16 to 22) | 18 (16 to 23) | 17.5 (15 to 21) | 0.413 |
| Pack-year ; Md (P25 to P75) | 11 (3 to 20) | 10 (5.4 to 20) | 8 (1.2 to 20) | 0.285 |
