# Supplementary material for: Smoking Is Associated with More Abdominal Fat in Morbidly Obese Patients
Source: PLoS One. 2015 May 15;10(5):e0126146. doi: 10.1371/journal.pone.0126146 (PMC4433108; doi:10.1371/journal.pone.0126146)
Supplement: S3 Table — Note: Adjusted for gender (except on gender stratum), age, diabetes, dyslipidemia, alcohol abuse and picky eater; b = regression coefficient; BMI = body mass index; WC = waist circumference; HC = hip circumference; WHR = waist to hip ratio; SMM = muscle mass; BMR = basal metabolic rate; values in bold = statistically significant (p<0.05). (DOCX) [file pone.0126146.s003.docx]

**S3 Table:**

| Outcomes | Total (n=83) | | |
| --- | --- | --- | --- |
|  | b | 95%CI | p |
| WC (cm) |  |  |  |
| Non-Adjusted | 0.87 | -1.08 to 2.82 | 0.377 |
| Adjusted | 0.79 | 0.97 to 2.54 | 0.375 |
| HC (cm) |  |  |  |
| Non-Adjusted | -0.08 | -1.59 to 1.44 | 0.920 |
| Adjusted | 1.21 | -0.38 to 2.81 | 0.134 |
| WHR |  |  |  |
| Non-Adjusted | -0.01 | -0.06to 0.03 | 0.613 |
| Adjusted | -0.01 | -0.06 to 0.05 | 0.828 |
| Basal Metabolic rate Kcal/dia |  |  |  |
| Non-Adjusted | 8.70 | -27.5 to 44.9 | 0.634 |
| Adjusted | 17.00 | -7.5 to 41.5 | 0.170 |
| Fat percentage % |  |  |  |
| Non-Adjusted | 0.07 | 0.43 to 0.57 | 0.787 |
| Adjusted | 0.37 | 0.12 to 0.86 | 0.136 |
| SMM (kg) |  |  |  |
| Non-Adjusted | -0.15 | -1.21 to 0.91 | 0781 |
| Adjusted | 0.16 | 0.71 to 1.03 | 0.712 |
